# Fabrication of Bowl Array Surface-Enhanced Raman Scattering Substrates via Ag Nanoparticle Self-Assembly on Polymer UV-Imprinted Microbowls for Enhanced Raman Detection of Microplastics

**DOI:** 10.3390/polym17212930

**Published:** 2025-10-31

**Authors:** Yihong Liu, Longchao Qi, Kaibo Guo, Xianlong Ning, Yiming Huang, Xun Lu

**Affiliations:** Department of Mechanical Engineering, Yanbian University, Yanji 133002, China; 2022010087@ybu.edu.cn (Y.L.); 2023010094@ybu.edu.cn (L.Q.); 2023010088@ybu.edu.cn (K.G.); 2023010093@ybu.edu.cn (X.N.); 2024010102@ybu.edu.cn (Y.H.)

**Keywords:** microscale bowl array structure, light-trapping effect, localized surface plasmon resonance effect, surface-enhanced Raman scattering effect, microplastic detection, cost-effective

## Abstract

A facile, efficient, and cost-effective strategy for fabricating a bowl array SERS (surface-enhanced Raman scattering) substrate is presented. The resulting substrate is dimensionally compatible with micrometer-sized microplastics and integrates both SERS enhancement and light-trapping effects, enabling highly sensitive detection of micrometer-sized microplastics. Initially, a pillar array template was produced via UV lithography, followed by UV imprinting to replicate bowl arrays with a diameter of 50 μm, a depth of 25 μm, and a periodicity of 100 μm. A gold layer was subsequently deposited, followed by the modification of its surface with AgNPs to construct the SERS substrate. The experimental results reveal that the optimal enhancement was achieved at an AgNP suspension concentration of 15 mg/mL. The substrate exhibited a detection limit of 10^−9^ M for rhodamine 6G with an enhancement factor (EF) of 2.02 × 10^7^ and successfully detected polyethylene (PE) microplastics of 5, 10, and 20 μm at concentrations down to 100 μg/mL, demonstrating outstanding sensing performance.

## 1. Introduction

Microplastics, as an emerging pollutant, have been widely detected in various environmental media including marine [1], soil [2], and atmospheric environments [3,4,5]. Studies have shown that microplastics can enter the human body through the food chain, drinking water, and even air [6,7], and accumulate in organs such as the liver [8], kidneys [9], and brain [10], inducing inflammatory responses [11], immune dysfunction [12], neurological damage [13], etc. Therefore, efficient and accurate detection of trace microplastics is of great importance for safeguarding human health.

Common microplastic detection methods, such as fluorescence dye labeling [14,15], optical microscopy [16], and scanning/transmission electron microscopy (SEM/TEM-EDS) [17], allow for rapid screening and morphological observation but lack chemical specificity and quantitative capability. Fourier-transform infrared spectroscopy can identify various polymers but is limited by spatial resolution and requires lengthy sample preparation [18]. Thermal analysis techniques, including pyrolysis–gas chromatography–mass spectrometry [19], thermogravimetric analysis (TGA) [20,21], and differential scanning calorimetry (DSC) [22], provide molecular-level polymer composition and thermal decomposition data, yet are time-consuming and equipment-intensive. In contrast, Raman spectroscopy offers high spatial resolution and in situ detection, enabling rapid, sensitive qualitative and quantitative analysis of microplastics, thus presenting unique advantages for microplastic research [9,23]. This technique is based on the fact that the characteristic chemical bonds of microplastics produce corresponding peaks under Raman spectral scanning and has been widely applied for the identification of various microplastics [24,25]. Shiwani S. et al. [26] proposed a High-Throughput Screening Raman Spectroscopy platform that integrates a 3.15 × 2.10 mm^2^ field of view with 1.4 µm spatial resolution to achieve rapid, label-free detection and classification of microplastics of various sizes. Validation using reference microplastic mixtures showed that the platform can accurately detect particles in the 7–400 µm range and offers robust morphological and chemical characterization capabilities. However, because the intrinsic Raman scattering intensity is extremely weak (about 10^−6^–10^−9^ of the incident light intensity), accurate quantitative measurements at trace levels remain challenging. By fabricating and applying SERS (surface-enhanced Raman scattering) substrates, Raman scattering intensity can be effectively enhanced, thereby significantly improving the performance of SERS in quantitative detection and analysis [27,28,29,30]. For example, Li et al. [31] developed a novel honeycomb-like AgNPs@TiO_2_ array SERS sensor, in which plasmonic silver nanoparticles (AgNPs) were uniformly anchored within periodic TiO_2_ nanocage arrays to form an AgNPs@TiO_2_ hybrid structure. The dual enhancement mechanisms of this architecture significantly increased the sensor’s sensitivity, enabling quantitative analysis of polystyrene microplastics in tap water, lake water, soil water, and seawater with detection limits of 100, 100, 100, and 250 μg/mL, respectively, demonstrating excellent practical applicability. Therefore, exploring efficient enhancement strategies to fabricate high-performance SERS substrate sensors is of great significance for strengthening Raman scattering and improving the qualitative and quantitative analysis of trace microplastic detection.

Surface-enhanced Raman scattering (SERS) substrate sensors typically exploit the localized surface plasmon resonance (LSPR) effect of metallic nanostructures, significantly amplifying the local electromagnetic field, thereby enhancing the Raman signal [32,33]. Zhu et al. fabricated a ZnO nanorod array with a dragonfly-wing-like architecture on polydimethylsiloxane (PDMS) and decorated it with AgNPs. This hybrid structure served as both a SERS and photo-induced enhanced Raman scattering substrate, effectively enabling trace analysis of microplastics. Coupled with a portable Raman device, this sensor successfully detected polyethylene (PE) and polystyrene microplastics at concentrations as low as 25 μg/mL [34]. Similarly, Quang Trung Lê et al. introduced electromagnetic hotspots at nanoparticle tips to construct anisotropic nano-STAR dimers embedded in a nanoporous substrate, enabling efficient identification of submicron plastic spheres. The method achieved a detection limit of 0.005% (≈50 ppm) for 0.005–4 μm particles without pretreatment, offering high sensitivity and rapid detection [35]. Additionally, SERS substrate sensitivity can also be enhanced through the design of specialized optical structures. Optical microcavity structures exhibiting the light-trapping effect have attracted considerable attention due to their high light utilization efficiency and low sample consumption [36,37]. This effect arises from multiple reflections and scattering of light at the bottom and inner walls of the microcavity, enabling efficient light absorption and localization, which greatly amplifies the electromagnetic field on the SERS substrate surface [38,39,40]. Lin et al. replicated negative pyramid microarrays from photolithographically and wet-etched silicon templates using PDMS and deposited a silver layer on the surface via thermal evaporation to construct a flexible SERS substrate. Multiple reflections of the incident laser within the negative pyramid microarrays enhanced the SERS signal approximately fourfold compared to flat substrates, achieving detection limits for rhodamine 6G (R6G) and malachite green below 10^−8^ M and 10^−9^ M, respectively [41]. These studies provide a solid foundation for highly sensitive Raman sensing. However, the fabrication of nanostructured and optical microcavity SERS substrates generally involves high development costs and complex processes. Moreover, microplastic particles are generally on the micrometer scale, substantially exceeding the dimensions of the nanostructures or microcavities, which hampers their efficient capture and limits the effective exploitation of the SERS effect and the light-trapping effect. Therefore, developing SERS sensors that are cost-effective, easy to operate, highly sensitive, and capable of accommodating the size of microplastics is of great importance for achieving efficient and accurate qualitative analysis of microplastics.

In this study, to address the issues of low utilization of the SERS effect and the structural size mismatch in microplastic detection, a convenient and cost-effective photolithographic dry film templating method was employed to fabricate a microscale bowl-shaped array SERS substrate. The structural design of the substrate enables a synergistic enhancement between the SERS effect and the light-trapping effect, thereby significantly improving detection sensitivity. First, a UV photolithography system equipped with a patterned photomask was set up, and exposure and development parameters were optimized to fabricate a pillar array template. Subsequently, the pillar array template was replicated into a bowl array substrate using UV imprinting. To generate a high-density electromagnetic field for Raman enhancement, a gold layer was deposited on the substrate surface via vacuum physical vapor deposition (PVD), followed by modification with AgNPs inside the microbowls, successfully constructing the bowl array SERS substrate. To evaluate the influence of AgNP coverage density on Raman enhancement performance, the relationship between the AgNP suspension concentration and particle coverage ratio was quantitatively analyzed. Comparative Raman measurements of R6G on different substrates were then conducted to determine the optimal AgNP concentration for substrate modification. Finally, the prepared substrate was validated for high sensitivity in the detection of low-concentration analytes using R6G and applied to the detection of PE microplastics.

## 2. Fabrication of Microscale Bowl-Shaped Array SERS Substrates

**Figure 1:**
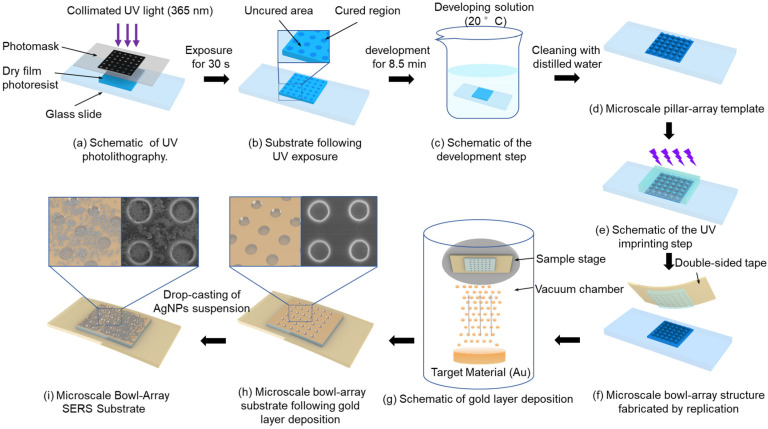
Schematic of fabrication process of bowl array SERS substrate.

This study integrates the existing theories and techniques of UV photolithography, development, and imprinting and independently designs and constructs a fabrication platform, establishing an efficient and convenient strategy for producing microscale bowl-shaped arrays. First, to fabricate the pillar array structure as the imprinting template for the bowl array, a UV direct-write exposure system was established, as illustrated in Figure 1a. In this setup, a collimated UV light beam passed through the photomask to expose the dry film photoresist. Under UV illumination through the circular array openings, the corresponding regions of the dry film photoresist were cured, as shown in Figure 1b. Subsequently, the exposed dry film photoresist was immersed in the developer solution (20 ± 0.5 °C) to dissolve the uncured regions, as illustrated in Figure 1c. After development and cleaning with distilled water, a pillar array template was obtained, as shown in Figure 1d. Finally, to increase template hardness and enable repeated use, the template underwent deep curing under a high-intensity UV light for 10 s.

The prepared template was replicated into bowl array structures using UV imprinting. Specifically, a layer of the photoresist was dropped onto the micron-scale pillar array template, and an anti-adhesive plastic sheet was placed on top. Appropriate pressure was applied to ensure that the photoresist fully filled the gaps of the pillar array and expelled air, as illustrated in Figure 1e. Subsequently, the photoresist was partially cured under UV illumination at 350 mW/cm^2^ while different pressures (0, 20, 40, and 60 N) were applied for 5 s. The photoresist layer was then separated from the pillar array template using double-sided tape instead of a plastic sheet, yielding a photoresist substrate with a bowl array structure, as shown in Figure 1f.

After the fabrication of the bowl array substrate, further modification with a gold layer and AgNPs was carried out to effectively enhance the SERS effect. During PVD of the gold layer, the working pressure was maintained at 0.08 mbar and the current at 41 mA, with a deposition rate of 0.55 nm/s, ensuring continuous growth of gold nanoparticles along the bowl-shaped surface to obtain a substrate uniformly covered with a dense gold layer. Subsequently, an evaporation-induced self-assembly strategy was employed to decorate the gold-coated substrate with AgNPs. Commercial AgNPs (average diameter 20 nm, standard deviation ± 3 nm) were dispersed in anhydrous ethanol containing 1.5 wt% polyvinylpyrrolidone (PVP), preparing suspensions with concentrations of 5, 10, 15, 20, 25, and 30 mg/mL. PVP acted as a dispersant to effectively suppress AgNP aggregation and maintain uniform dispersion in the suspension. Each suspension was homogenized using a programmable mixer (1500 rpm, 2 min) and then treated in an ultrasonic bath (40 kHz, 200 W, 10 min) to achieve solid–liquid uniformity. A 10 μL droplet of each suspension was deposited onto the bowl array SERS substrate using a micropipette and left to dry at room temperature until complete solvent evaporation, forming a coupled AgNP–Au layer composite structure on the substrate, as shown in Figure 1i.

**Figure 2:**
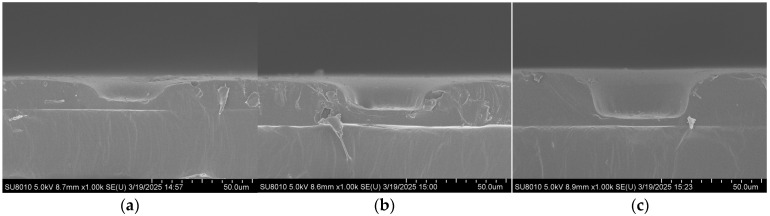
SEM cross-sectional images of the microscale bowl structure under different pressures: (**a**) 0 N; (**b**) 20 N; (**c**) 40 N.

Scanning electron microscopy (FE-SEM, SU8010, Hitachi, Tokyo, Japan) was employed to characterize the substrates, and the effect of imprinting pressure during the UV imprinting process on the structure’s morphology was investigated. As shown in Figure 2, UV imprinting was performed under four different pressures (0, 20, 40, and 60 N), and the depth of the bowl-shaped structures gradually increased with increasing pressure. At 0 and 20 N, the structures were relatively shallow, indicating that the pressure was insufficient to fully replicate the bowl shape. At 40 N, well-defined bowl-shaped structures were obtained, with a sufficient thickness of the underlying photoresist layer to maintain substrate strength and prevent structural damage during replication. Therefore, 40 N was determined to be the optimal imprinting pressure for UV imprinting replication.

## 3. Effect of AgNP Coverage Morphology on Substrate Performance

**Figure 3:**
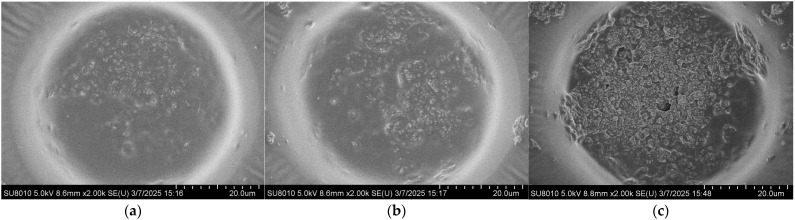

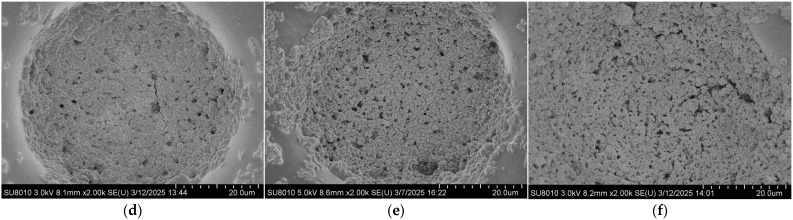
Surface morphologies of the substrate after drop-casting self-assembly AgNP suspensions with different concentrations: (**a**) 5 mg/mL; (**b**) 10 mg/mL; (**c**) 15 mg/mL; (**d**) 20 mg/mL; (**e**) 25 mg/mL; (**f**) 30 mg/mL.

The coverage morphology of AgNPs is closely related to the Raman signal enhancement of the substrate. To further improve substrate performance and surface morphology, the density of AgNPs on the substrate after self-assembly was controlled by adjusting the concentration of the AgNP suspension. The SERS performance of the substrate was evaluated using the Raman signal intensity of R6G. Specifically, 10 μL droplets of 10^−5^ M R6G ethanol solution were deposited onto the substrate using a micropipette. After the droplets had completely evaporated, Raman measurements were performed with a portable Raman spectrometer (ATR3110, OPTOSKY, Xiamen, China). Meanwhile, the surface morphology of substrates coated with AgNPs at different suspension concentrations was characterized by using a field emission scanning electron microscope, as shown in Figure 3. It can be observed that at a concentration of 5 mg/mL, the AgNPs are sparsely distributed on the surface, resulting in limited coverage of the gold layer and extensive exposed regions. As the concentration increases, the surface coverage of AgNPs gradually improves. When the concentration reaches 15 mg/mL, the area occupied by AgNPs exceeds that of the exposed gold layer. At higher concentrations of 20–30 mg/mL, the gold surface becomes almost completely covered by AgNPs.

**Figure 4:**
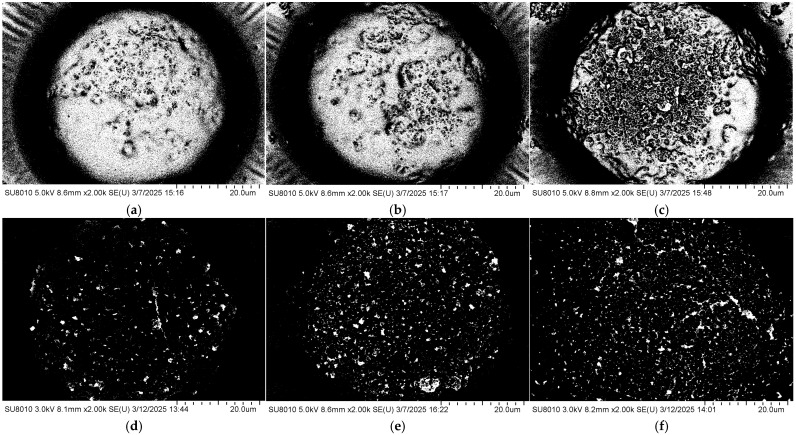
SEM images of the bowl structures processed using ImageJ at different AgNP suspension concentrations: (**a**) 5 mg/mL; (**b**) 10 mg/mL; (**c**) 15 mg/mL; (**d**) 20 mg/mL; (**e**) 25 mg/mL; (**f**) 30 mg/mL.

**Figure 5:**
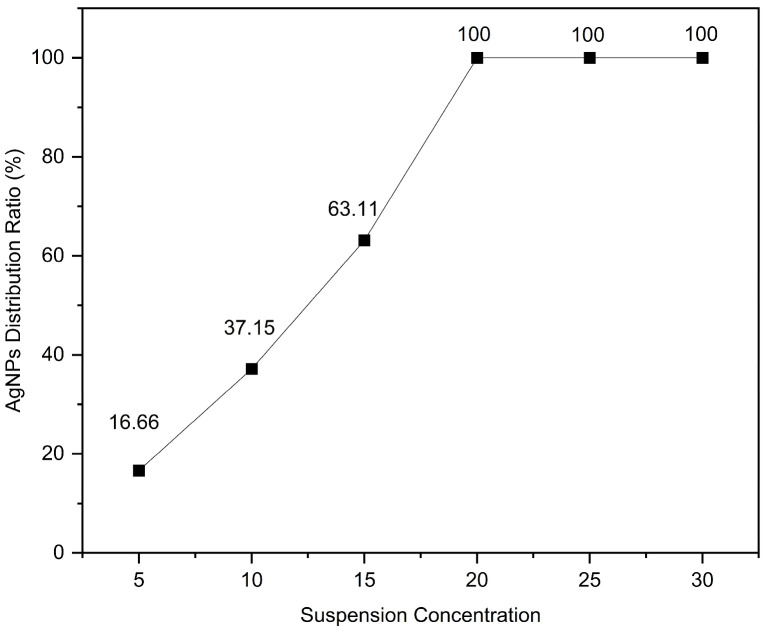
The relationship between the coverage ratio of AgNPs and the suspension concentration.

The coverage ratio of silver nanoparticles (AgNPs) on the gold layer significantly affects the electromagnetic coupling strength at the Au–Ag contact regions, thereby determining the overall performance of the substrate. To quantitatively analyze this relationship, the SEM images shown in Figure 3 were processed using ImageJ v1.54g to calculate the coverage ratio of AgNPs within the bowl-shaped structures, and the corresponding relationship between the concentration of the AgNP suspension and the particle coverage ratio was established. The ImageJ-processed images are presented in Figure 4, and the calculated coverage ratios are summarized in Figure 5. The results indicate that with increasing AgNP suspension concentration, particle coverage on the substrate surface gradually increased. Specifically, at suspension concentrations of 5, 10, 15, 20, 25, and 30 mg/mL, the corresponding AgNP coverage ratios were 16.66%, 37.15%, 63.11%, 100%, 100%, and 100%, respectively.

**Figure 6:**
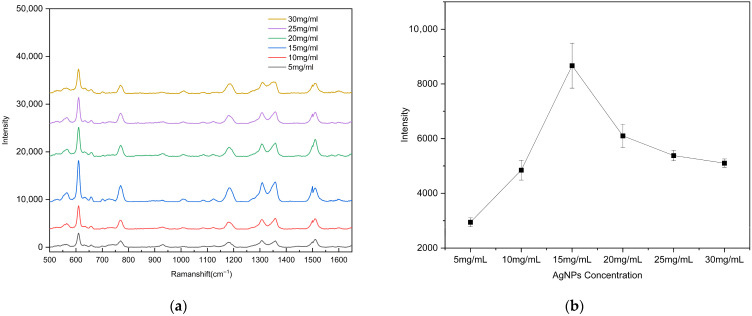
(**a**) Raman spectra of R6G (10^−5^ M) as a function of the AgNP suspension concentration used for drop-coating on the substrate; (**b**) calibration curves of the vibrational band intensities at ~612 cm^−1^. The error bars represent the standard deviations of three measurements.

To evaluate the surface-enhanced Raman scattering (SERS) performance of the substrates with different AgNP coverage ratios, the 10^−5^ M rhodamine 6G (R6G) solution was drop onto each substrate prepared with different AgNP concentrations. Raman detection parameters: excitation wavelength 785 nm, integration time 1 s, laser power 50 mW, spectral range 500–1650 cm^−1^). After natural drying, Raman spectra were recorded using a portable Raman spectrometer. To ensure data reliability, spectra were collected from three different bowl-shaped regions on each substrate. As shown in Figure 6, the substrate fabricated using an AgNP suspension concentration of 15 mg/mL (coverage ratio of 63.11%) exhibited the strongest enhancement of the R6G Raman signal. When the AgNP concentration was low (5 or 10 mg/mL), the sparse particle coverage failed to generate a sufficiently dense and strong localized electromagnetic field. In contrast, at concentrations above 20 mg/mL, the AgNPs almost completely covered the gold surface, resulting in a reduction in nanoscale gaps and Au–Ag junction regions, which weakened electromagnetic coupling and diminished the enhancement effect. Overall, an AgNP suspension concentration of 15 mg/mL yielded the optimal particle coverage and Raman signal enhancement performance.

## 4. Sensitivity Evaluation of Bowl Array SERS Substrates

### 4.1. R6G Detection on Bowl Array SERS Substrates

**Figure 7:**
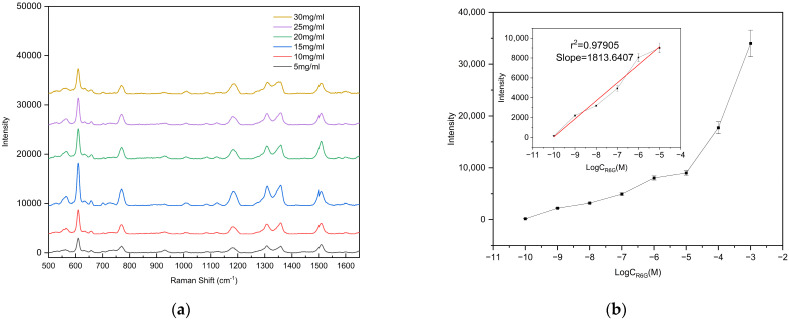
(**a**) Raman spectra of R6G with concentrations ranging from 10^−3^ to 10^−10^ M; (**b**) calibration curve of the vibrational band intensity at 612 cm^−1^. The error bars represent the standard deviations obtained from three independent measurements.

To evaluate the detection capability of the fabricated substrate for low-concentration analytes, different concentrations of R6G solutions were prepared for limit-of-detection (LOD) analysis. Specifically, 47.091 mg of solid R6G was dissolved in 1 mL of 99.99% anhydrous ethanol under ultrasonication until fully dissolved, yielding a 0.1 M R6G ethanol stock solution. The stock solution was then serially diluted to concentrations down to 10^−10^ M, and all prepared solutions were stored in the dark at low temperature until use. For measurement, 10 μL of each R6G solution at different concentrations was dropped onto the center of the substrate, and Raman spectra were collected after complete evaporation of the droplet. To ensure the reliability and representativeness of the results, Raman spectra were collected from three different bowl-shaped structures on each substrate. (ATR3110, OPTOSKY; excitation wavelength 785 nm, integration time 1 s, laser power 50 mW, spectral range 500–1650 cm^−1^), as shown in Figure 7. The results demonstrate that the Raman signal intensity gradually decreased with decreasing R6G concentration. Remarkably, even at a concentration as low as 10^−9^ M, the characteristic peak at 610 cm^−1^ could still be clearly detected, whereas no characteristic peaks were observed when the concentration was further reduced to 10^−10^ M. Therefore, the detection limit of the fabricated substrate for R6G was determined to be 10^−9^ M. Supplementary Figure S1a confirms that the peak at 610 cm^−1^ originates from R6G.

In SERS research, the enhancement factor (EF) is a crucial parameter to evaluate the intensity of the SERS effect. The EF was calculated using Equation (1), where I_SERS_ and I_NR_ represent the Raman signal of R6G on the prepared SERS substrate and the silicon substrate, respectively, and C_SERS_ and C_NR_ are the concentrations of R6G deposited on the prepared substrate and the silicon substrate, respectively. According to Equation (1), the enhancement factor of the prepared SERS substrate was calculated to be 2.02 × 10^7^, demonstrating that the fabricated bowl array SERS substrate exhibits excellent performance for detecting low-concentration targets.

(1) $$EF=\frac{I_{SERS}\times C_{NR}}{I_{NR}\times C_{SERS}}$$

### 4.2. Microplastic (PE) Detection on Bowl Array SERS Substrates

**Figure 8:**
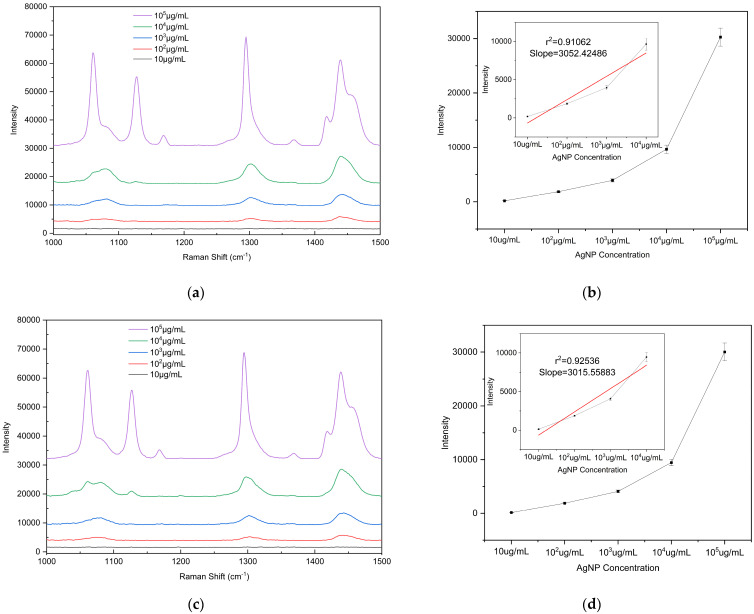

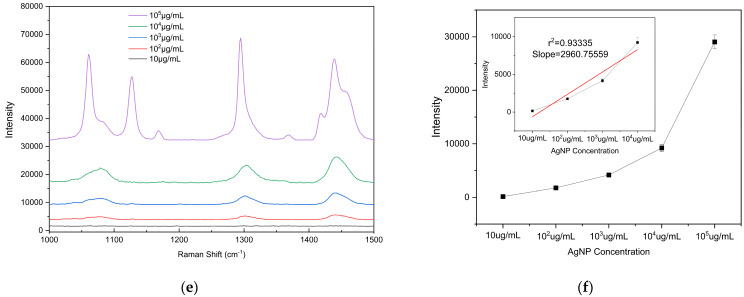
Raman spectra of PE microplastics with different particle sizes and intensity plots as a function of concentration: (**a**,**b**) 5 µm, (**c**,**d**) 10 µm, and (**e**,**f**) 20 µm. The calibration curve of the vibrational band intensity at 1440 cm^−1^. Error bars indicate standard deviations of three measurements.

To evaluate the detection sensitivity of the prepared substrate toward microplastics, PE particles with diameters of 5, 10, and 20 μm were used as analytes to determine the LODs on the substrate. The PE particles were mixed with anhydrous ethanol (purity ≥ 99.8%) at a mass-to-volume ratio of 1:100 (i.e., 10^5^ μg/mL) and dispersed via ultrasonic treatment (40 kHz, 100 W, 10 min) to form a homogeneous suspension. The initial suspension was then serially diluted tenfold to prepare solutions with different concentrations ranging from 10^5^ μg/mL to 10 μg/mL for subsequent measurements. A 2 μL aliquot of the microplastic suspension (initial concentration 10^5^ μg/mL) was uniformly dropped onto the substrate surface. After drying at room temperature, the Raman signal at this location was measured using a portable Raman spectrometer, and substrate performance was evaluated based on the characteristic peak intensity. To ensure the reliability of the results, Raman spectra were measured at three different bowl structures on the substrate. Raman detection parameters: excitation wavelength 785 nm, laser power 150 mW, integration time 25 s, detection range 1000–1500 cm^−1^). The drop-casting self-assembly and measurement procedure was repeated for each suspension concentration to determine the detection limit. According to literature reports and the spectra shown in Figure 8, the characteristic Raman peaks of PE microplastics are located at 1063 cm^−1^, 1128 cm^−1^, 1295 cm^−1^, and 1440 cm^−1^ [42,43]. Among these, the peak at 1440 cm^−1^ (C–H_2_ bending vibration) is the most pronounced and readily identifiable across different concentrations and was therefore selected as the criterion for PE Raman detection. Supplementary Figure S1b confirms that the peak at 1440 cm^−1^ originates from PE microplastics. Comparison of the 1440 cm^−1^ peak shows that the Raman signal intensity is positively correlated with the concentration of PE suspensions of all three particle sizes, with a detection limit of 10^2^ μg/mL. These results indicate that the fabricated bowl array SERS substrate exhibits excellent potential for microplastic detection.

## 5. Conclusions

In the detection of micrometer-sized microplastics, a major challenge faced by conventional SERS substrates is the relatively large size of the target particles. Traditional nanostructures exhibiting LSPR are often unable to effectively interact with such particles, thereby limiting signal enhancement. A bowl array structure was designed to achieve effective size matching with microplastic particles, enabling the localized surface plasmon resonance of silver nanoparticles (AgNPs) to fully interact with the targets. Additionally, the micrometer-sized bowl structures function as optical microcavities, providing strong light-trapping effects that further enhance substrate sensitivity.

Initially, a pillar array was fabricated using ultraviolet (UV) direct-write lithography. This array served as a template for UV nanoimprinting replication of the bowl array SERS substrates. Following the deposition of a gold layer and subsequent modification with AgNPs, the bowl array SERS substrate was successfully constructed. Experimental optimization revealed that an AgNP suspension concentration of 15 mg/mL yielded the strongest Raman signal enhancement. Leveraging the synergistic enhancement effect of the Au–Ag bimetallic system alongside the unique light-trapping mechanism of the bowl structures, high-sensitivity detection of both R6G and PE microplastic particles was achieved. Limit of detection experiments demonstrated that the optimized bowl array SERS substrate could detect R6G at concentrations as low as 10^−9^ M and PE microplastics down to 100 μg/mL, exhibiting excellent analytical performance. These results indicate that the substrate has significant potential for environmental monitoring, particularly for convenient and efficient detection of micrometer-sized plastics.

Although this study achieved high sensitivity and facile fabrication, there remains room for further optimization of the substrate structure and materials. Future research may focus on tailoring the microstructural geometry and dimensions of the bowls, as well as enhancing their optical microcavity properties, to achieve better size matching with microplastics of various dimensions and superior light-trapping efficiency, thereby further improving the detection performance of the substrate.

## Data Availability

The original contributions presented in this study are included in the article/Supplementary Materials. Further inquiries can be directed to the corresponding author.

## Supplementary Materials

The following supporting information can be downloaded at: https://www.mdpi.com/article/10.3390/polym17212930/s1, Figure S1: Stacked Raman spectra of R6G/PE, anhydrous ethanol, PVP, and the bare substrate. (a) Spectral range for R6G detection; (b) Spectral range for PE detection.

## Abbreviations

The following abbreviations are used in this manuscript:

SERS	Surface-enhanced Raman scattering
AgNPs	Silver nanoparticles
PDMS	Polydimethylsiloxan
PE	Polyethylene
PVD	Physical vapor deposition
R6G	Rhodamine 6G
PVP	Polyvinylpyrrolidone
SEM	Scanning electron microscopy
LOD	Limit-of-detection
EF	Enhancement factor
