# Supplementary material for: Fabrication of Bowl Array Surface-Enhanced Raman Scattering Substrates via Ag Nanoparticle Self-Assembly on Polymer UV-Imprinted Microbowls for Enhanced Raman Detection of Microplastics
Source: Polymers (Basel). 2025 Oct 31;17(21):2930. doi: 10.3390/polym17212930 (PMC12608143; doi:10.3390/polym17212930)
Supplement: Supplementary file 1 [file polymers-17-02930-s001.zip › polymers-3941929-supplementary.pdf]

polymers

MDPI

Supplementary Information

Fabrication of Bowl Array Surface-Enhanced Raman Scattering Substrates via Ag Nanoparticle Self-Assembly on Polymer UV-imprinted Microbowls for Enhanced Raman Detection of Microplastics

Yihong Liu [1,†], Longchao Qi [1,†], Kaibo Guo [1], Xianlong Ning [1], Yiming Huang [1] and Xun Lu [1*]

[1] Department of Mechanical Engineering, Yanbian University, Yanji 133002, China
† These authors contributed equally to this work.
* Correspondence: luxun@ybu.edu.cn

S1. Analysis of the Origin of Raman Peaks

Since the AgNPs were stabilized by PVP and all analytes were dissolved in ethanol, it was essential to verify that the observed SERS bands originated from the vibrational modes of the analytes themselves rather than from the stabilizer molecules and/or solvent. To ensure this, we compiled the Raman spectra of PVP and completely evaporated ethanol measured under identical parameters, and compared them with the Raman spectra of R6G and microplastics on the substrate, as shown in Figures S1(a) and (b). The results demonstrate that no additional bands appeared from PVP or the solvent ethanol beyond the intrinsic spectral features of the substrate. In contrast, characteristic peaks unique to the target analytes emerged only when R6G and microplastics were present on the substrate. This study primarily focused on the characteristic peaks at 610 cm^{-1} in Figure S1(a) and 1440 cm^{-1} in Figure S1(b). It is evident that these characteristic peaks originate exclusively from the analytes, confirming that the SERS bands detected at these positions are not attributed to vibrational modes of the stabilizer molecules and/or solvent.

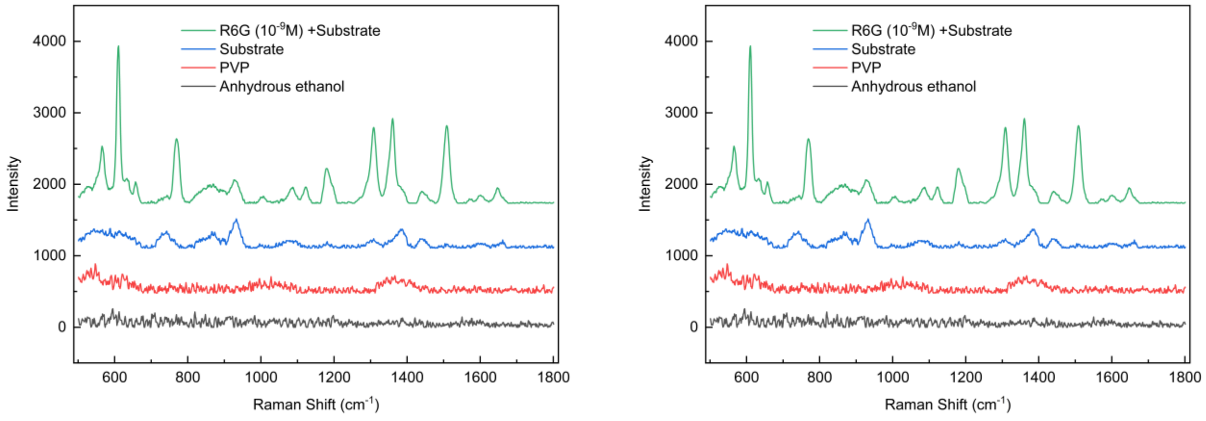

(a) (b)

Figure S1. Stacked Raman spectra of R6G/PE, anhydrous ethanol, PVP, and the bare substrate. (a) Spectral range for R6G detection; (b) Spectral range for PE detection.
